# Is Prophylactic Therapy for Varices Justified? Can the First Variceal Bleed be Predicted?

**DOI:** 10.1155/1989/68458

**Published:** 1989

**Authors:** G. W. Johnston

**Affiliations:** Royal Victoria Hospital Grosvenor Road Belfast BT12 6BA Ireland


### IS PROPHYLACTIC THERAPY FOR VARICES JUSTIFIED? CAN THE FIRST VARICEAL BLEED BE PREDICTED?

### ABSTRACT

*The North Italian Endoscopic Club for the Study and Treatment of Esophageal Varices.* (1988) Prediction of the first variceal hemorrhage in patients with cirrhosis of the liver and esophageal varices. A Prospective Multicenter Study. The New England Journal of Medicine 319, 983–989.

**We conducted a prospective study of 321 patients with cirrhosis of the liver and esophageal varices with no history of bleeding to see whether a comprehensive analysis of their clinical features and of the endoscopic appearances of their varices could help to identify those at highest risk for bleeding. Varices were classified endoscopically as suggested by the Japanese Research Society for Portal Hypertension. Patients were followed for 1 to 38 months (median, 23), during which 85 patients (26.5 percent) bled. Multiple regression analysis (Cox's model) revealed that the risk of bleeding was significantly related to the patient's modified Child class (an index of liver dysfunction based on serum albumin concentration, bilirubin level, prothrombin time, and the presence of ascites and encephalopathy), the size of the varices, and the presence of red wale markings (longitudinal dilated venules resembling whip marks) on the varices. A prognostic index based on these variables was devised that enabled us to identify a subset of patients with a one-year incidence of bleeding exceeding 65 percent. The index was prospectively validated on an independent sample of 75 patients with varices and no history of bleeding.**

**We conclude that our prognostic index, which identifies groups of patients with one-year probabilities of bleeding ranging from 6 to 76 percent, can be used to identify candidates for prophylactic treatment. (N Engl J Med 1988; 319;983–9)**

### PAPER DISCUSSION

In June 1979 the Japanese Research Society for Portal Hypertension met in Osaka and drew up rules for the endoscopic classification of varices according to size, shape, location, colour and colour signs[1]. Beppu and colleagues[2] retrospectively analysed their endoscopic recordings on 172 patients and looked at the relationship between endoscopic findings and the incidence of bleeding. On the basis of their results they proposed a complicated discriminant equation as a means of predicting the likelihood of non-bleeders becoming bleeders. However when the North Italian group applied the Japanese formula to their patients, they obtained a gross over-estimate of the risk of bleeding. Even high risk Japanese patients selected by Inokuchi[3] according to Beppu's criteria had a less than 20 per cent chance of bleeding at one year. Paquet[4] used endoscopic signs of impending haemorrhage together with coagulation deficiencies to select out high risk patients for his trial of prophylactic therapy. In the three year follow up period there was an exceptionally high rate of bleeding in the control patients; two thirds experienced haemorrhage. Witzel and his colleagues[5] reported that 57 per cent of their control patients bled in a twenty-five month period and their patients had not been selected because of high risk. The incidence of bleeding ranged from 35 per cent for small varices to 53 per cent for medium varices and 83 per cent for large varices. In Koch and colleagues'[6] propylactic trial they again took all comers with varices and in the three year follow up period only 30 per cent of their patients in the control arm bled. This figure is much closer to that reported in the United Kingdom by Hayes and colleagues[7], who found an 18 per cent incidence of bleeding in a follow up period of almost two years. The figure for the Italian study is 26 per cent for a median follow up period of 23 months. Thus if only approximately 25 per cent are likely to survive the first haemorrhage, prophylactic therapy is likely to benefit less than 10 per cent of all patients.

The trials of propylactic shunts in the 1960's failed to demonstrate an improvement in survival for operated patients. However these trials suffered from the fact that the clinicians had no means of selecting for the trial only those patients with a high risk of bleeding. Thus in the control group only about 30 per cent of the patients actually bled. In addition there was a significant initial operative mortality for shunt surgery. The development of formulae for identifying patients at high risk of bleeding together with the availability of sclerotherapy with its low mortality in the non bleeding patient, has rekindled interest in prophylactic therapy. Harold Conn[8] pointed out that "as with a volcano, it is difficult to know when a varix will erupt". However it would be useful if we could estimate the percentage risk of rupture for an individual patient during the ensuing couple of years. The North Italian Endoscopic Club (N.I.E.C.) for the study and treatment of oesophageal varices set out to see whether endoscopy alone or in combination with clinical and biochemical data could recognise prospectively patients with the highest risk of bleeding and hence the ones who would benefit most from prophylactic therapy. In their introduction they refer to a "40 per cent chance of dying of the initial bleeding episode" and quote in evidence four references dating back as far as 1968; none of the series referred to had used sclerotherapy. In most modern series sclerotherapy controls bleeding in over 90 per cent of patients with a hospital mortality usually well under 30 per cent[9,10,11,12]. The possible use of prophylactic therapy must be seen against the background of these more relevant figures. One would have to be able to identify a subset of patients with a high risk of bleeding and then put them into a controlled trial which offers the best available form of therapy to patients in the control group who subsequently bleed. The problem of the early trials which advocated prophylactic sclerotherapy was that this was not done and therefore the mortality in the control patients was unacceptably high[4,5]. Subsequent trials of prophylactic sclerotherapy have been less enthusiastic[13,14]. The International Symposium on Prophylaxis of Variceal Bleeding held in Munich in 1986 concluded that there was no justification for prophylactic therapy other than in controlled trials[15].

This multicentre study by the North Italian Endoscopic Club suggests that their prognostic index could be used to identify candidates suitable for prophylactic therapy. It enabled them to "identify a subset of patients with a one year incidence of bleeding exceeding 65 per cent". However this subset was only 11 patients out of 321 studied (3.4 per cent), and they admit that using their formula "the number of very high risk patients who can be identified with it is very small". Of the whole series of 321 patients, 85 (26 per cent) developed upper gastro-intestinal bleeding during a median follow up of 23 months. This represents 0.01 haemorrhage per patient per month. Of the 85 who bled, only 53 had proven variceal bleeding. In the results section, the paper states that "in the remaining 28 patients endocopy either could not be performed, or was performed after bleeding had stopped", although in the discussion it states that no endoscopy was performed on these 28 patients. They assume that the majority of these non-endoscoped patients bled from varices and take the liberty of including them in the percentage rates of bleeding given in Table 6. Since the point of the exercise is to identify patients that would benefit from prophylactic therapy for varices, it is somewhat unsatisfactory to include a third of the patients with no diagnosis of the source of bleeding.

In their initial assessment the authors used Beppu's endoscopic scoring system together with a number of clinical and biochemical variables. Following a series of complicated mathematical and statistical calculations, they reduced the number of variables considered most useful to three, namely, Child's classification, variceal size (three categories) and red wale markings (four categories). The advantage of using these three variables is that they are simple, commonly used and less liable to observor error than many of the criteria employed in the Japanese classification. Their "pocket chart" gives an easy method of calculating the N.I.E.C. prognostic index on the basis of scores obtained for the three variables. They then reduced the rather complicated six-risk groups of Beppu's classification down to three in a prospective study of a further 75 patients. Of these 75 patients, 19 (25 per cent) bled during "a short follow up". Where the N.I.E.C. index was less than 30, five of the 36 patients (14 per cent) bled, between 30 and 36 seven out of 20 bled (35 per cent) bled and where the N.I.E.C. was greater than 36, seven out of 19 (37 per cent) bled. The actual rate of bleeding in these patients studied prospectively was almost identical to that predicted from the retrospective study. Of the 75 patients studied prospectively less than 20 per cent scored over 30 on the N.I.E.C. index. This small subset of patients are the ones most likely to benefit from prophylactic therapy but only a multicentre trial could provide sufficient numbers to give an answer. This present study suffers from being a multi-centre trial involving nine clinics and 56 clinicians. Of the original 383 patients, 36 were excluded for various reasons, leaving 343 to enter the study. However 26 patients failed to attend after the first endoscopy. The potential follow up period was 24 to 38 months (median 23 months) and yet a further 59 patients were lost to follow up inside the 23-month period.

The struggle to find a formula to predict patients at high risk is commendable but we have to agree with the authors' own conclusions that "The N.I.E.C. index is not ideal". Certainly if propylactic therapy is to be employed in the future, it will be necessary to have some easily reproducible method of identifying patients at high risk of variceal bleeding and this study provides the most useful formula to date.

**Keywords** Prophylactic therapy, oesophageal varices, bleeding risk

G.W. Johnston
Royal Victoria Hospital
Grosvenor Road
Belfast BT12 6BA
Northern Ireland

*References*

1. *Japanese Research Society for Portal Hypertension (1980) The General rules for recording endoscopic findings on esophageal varices. Japanese Journal of Surgery,* **10** 84–87.
2. Beppu K, Inokuchi K, Koyanagi N, Nakayama S, Sakata H, Kitano S, Kobayashi M (1981) Prediction of variceal hemorrhage by esophageal endoscopy. *Gastrointestinal Endoscopy,* **27**, 213–218.

3. Inokuchi K (1984) Prophylactic portal nondecompression surgery in patients with esophageal varices. *Annals of Surgery*, **200**, 61–65.
4. Paquet K J (1982) Prophylactic endoscopic sclerosing treatment of the esophageal wall in varices – a prospective controlled randomized trail. *Endoscopy*, **14**, 4–5.
5. Witzel L, Wolbergs E, Merki H (1985) Prophylactic endoscopic sclerotherapy of oesophageal varices: a prospective controlled study. *Lancet*, 773–775.
6. Koch H, Henning H, Grimm H, Soehendra N (1986) Prophylactic sclerosing of esophageal varices. Results of a prospective controlled trial. *Endoscopy*, **18**, 40–43.
7. Hayes P C, Westaby D, Williams R (1985) Prophylactic endoscopic sclerotherapy of oesophageal varices – letter. *Lancet i*, 1106.
8. Conn H O (1980) The varix-volcano connection. *Gastroenterology*, **79**, 1333–1337.
9. Terblanche J, Yakoob H I Bornman P C, Stiegmann G V, Bane R, Jonker M, Wright J, Kirsch R (1981) A five-year prospective evaluation of tamponade and sclerotherapy. *Annals of Surgery*, **4**, 521–529.
10. Westaby D, MacDougall B R D, Williams R (1985) Improved survival following injection sclerotherapy for esophageal varices; final analysis of a controlled trial. *Hepatology*, **5**, 827–830.
11. Paquet K J, Feussner H (1985) Endoscopic sclerosis and esophageal tamponade in acute haemorrhage from esophagogastric varices. A prospective controlled randomized trial. *Hepatology*, **5**, 580–583.
12. Spence R A J, Anderson J R, Johnston G W (1985) Twenty-five years of injection sclerotherapy for bleeding varices. *British Journal of Surgery*, **72**, 195–198.
13. Gregory P, Hartigan P, Amodeo D *et al* (1987) Propylactic sclerotherapy for esophageal varices in alcoholic liver disease; results of a V.A. co-operative randomized trial. *Gastroenterology*, **92**, 1414.
14. Trigger D R, Smart H L, Hosking S W, Johnson A G (1988) Controlled trial of prophylactic sclerotherapy of oesophageal varices in England: Interim analysis of 103 patients. *Gut* **28**, A1430–A1431.
15. Terblanche J (1986) Sclerotherapy for prophylaxis of variceal bleeding. *Lancet i.* 961–962.

### THE APPROACH TO CARCINOMA OF THE PROXIMAL HEPATIC DUCTS: MORE RADICAL OR MORE CONSERVATIVE

### ABSTRACT

*S. Bengmark, H Ekberg, A. Evander, B. Klofver-Stahl and K-G. Tranberg* (1988) Major liver resection for hilar cholangiocarcinoma. Ann. Surg. 207;120–125.

**Between 1968 and 1984 liver resection with curative attempt was performed in 22 patients with hilar cholangiocarcinoma. Right lobectomy was performed in 4 patients, extended right lobectomy in 7, left lobectomy in 8, and excision of the median segment of the left lobe (segment IV) in 3. Bilio-enteric continuity was restored by hepatocholedochostomy in 17 patients and hepatojejunostomy in 4. (One patient had external transhepatic catheter drainage and no internal bile drainage). Operative mortality rate was 27% and caused by excessive intraoperative bleeding, sepsis, or liver insufficiency. Postoperative complications occurred in 57% of patients surviving the operation and were due mainly to leakage from the hepatocholedochostomy. Median survival was 6 months, and one third of the patients survived 1 year. Three patients survived 10 years and were among the four patients in whom a tumor-free resection margin was obtained (one of them died in the postoperative phase). It is concluded that resection of hilar cholangiocarcinoma may give long-term survival if a free resection margin is obtained. The importance of a free resection margin indicates that surgery should be aggressive and include liver resection.**

